# Management of Idiopathic Pyoderma Gangrenosum With Azathioprine As the Primary Adjunct in an Asian Man: A Case Report

**DOI:** 10.7759/cureus.25177

**Published:** 2022-05-21

**Authors:** Alina Nazir, Ali Zafar

**Affiliations:** 1 Medicine, Jinnah Hospital, Allama Iqbal Medical College, Lahore, PAK

**Keywords:** idiopathic pyoderma gangrenosum, azathioprine, diagnostic dilemma, non-healing ulcers, pathergy test, cyclosporine

## Abstract

Pyoderma gangrenosum (PG) is a very rare, non-infectious, progressive inflammatory condition falling under the umbrella of neutrophilic dermatoses. It is an ulcerative condition with a wide variety of cutaneous manifestations and multiple clinical variants (classic ulcerative, pustular, bullous, and superficial granulomatous). Additionally, owing to similar patterns of mucocutaneous ulceration, it has certain overlaps with other neutrophilic diseases frequently observed in clinical practice. Pyoderma gangrenosum may occur in association with systemic conditions such as inflammatory bowel disease, hematological malignancies, or as a part of an inherited inflammatory syndrome. However, in rare cases, it may have an idiopathic origin as well. With no specific standardized diagnostic and treatment protocols in place, the management of pyoderma gangrenosum is primarily guided by pre-existing literature or is tailored according to the individual’s disease pattern, type, and associations. Currently, the pathophysiology of pyoderma gangrenosum remains elusive at best. All the aforementioned reasons contribute significantly to PG being labeled as a "diagnostic dilemma" or more commonly as a "diagnosis of exclusion" with frequent incidences of delayed diagnosis or misdiagnosis resulting in catastrophic delays in management.

A 35-year-old Asian male presented with bilateral painful, violaceous ulcers with undermined edges involving the shins for the past three months. Routine investigations carried out were indicative of an underlying infection owing to a raised leucocyte count. Discharge from the lesion, however, showed no evidence of microbial growth. The ulcer progressively increased in size, despite optimal wound care and empirical treatment. Skin biopsy demonstrated central necrosis and ulceration of the epidermis and dermis with neutrophilic infiltrates. Phenomenon of pathergy was demonstrated following the formation of a new ulcer at the site of intravenous cannulation during hospital admission. Additionally, aggravation of pre-existing ulcers following their debridement was also indicative of a positive pathergy test. Ultimately, the diagnosis of pyoderma gangrenosum was made upon the successful exclusion of all the other differential diagnoses. Presence of an associated systemic disease could not be appreciated, leading to it being labeled as a case of idiopathic pyoderma gangrenosum. Supportive treatment with non-adhesive, moist dressings was initiated along with topical tacrolimus (0.1%) application. Treatment modalities utilized were steroids and azathioprine in divided doses owing to contraindications to the traditional option of cyclosporine. The patient showed a rapid response to steroids and azathioprine. The ulcers healed with characteristic cribriform scarring within three months of initiation of treatment.

## Introduction

Pyoderma gangrenosum (PG), although first described nearly 100 years ago remains a diagnostic dilemma for clinicians worldwide. The underlying etiology of pyoderma gangrenosum has been extensively researched, however, it continues to present profound diagnostic and therapeutic challenges. Boasting a variety of differential diagnoses owing to its variable patterns of mucocutaneous ulceration, instances of delayed diagnosis and/or misdiagnosis are not uncommon in the case of pyoderma gangrenosum. A delayed diagnosis and/or misdiagnosis may prove to exert a detrimental effect on the patient's care and disease prognosis. Additionally, there are no specific guidelines for the diagnosis and treatment of PG, further adding to its reputation as a "diagnostic dilemma."

Pyoderma gangrenosum (PG) is often associated with certain gastrointestinal diseases such as inflammatory bowel disease (IBD), hematological malignancies, or inherited inflammatory syndromes. Rarely, PG may have an idiopathic origin. The aim of this study was to provide an overview of the challenges faced by clinicians in diagnosing and managing patients with PG. In addition, we aimed to demonstrate the efficacy of a relatively under-utilized immunosuppressant, i.e., azathioprine as a primary adjunct for the purpose of inducing remission in the case of an Asian man afflicted with the disease.

## Case presentation

**Figure 1:**
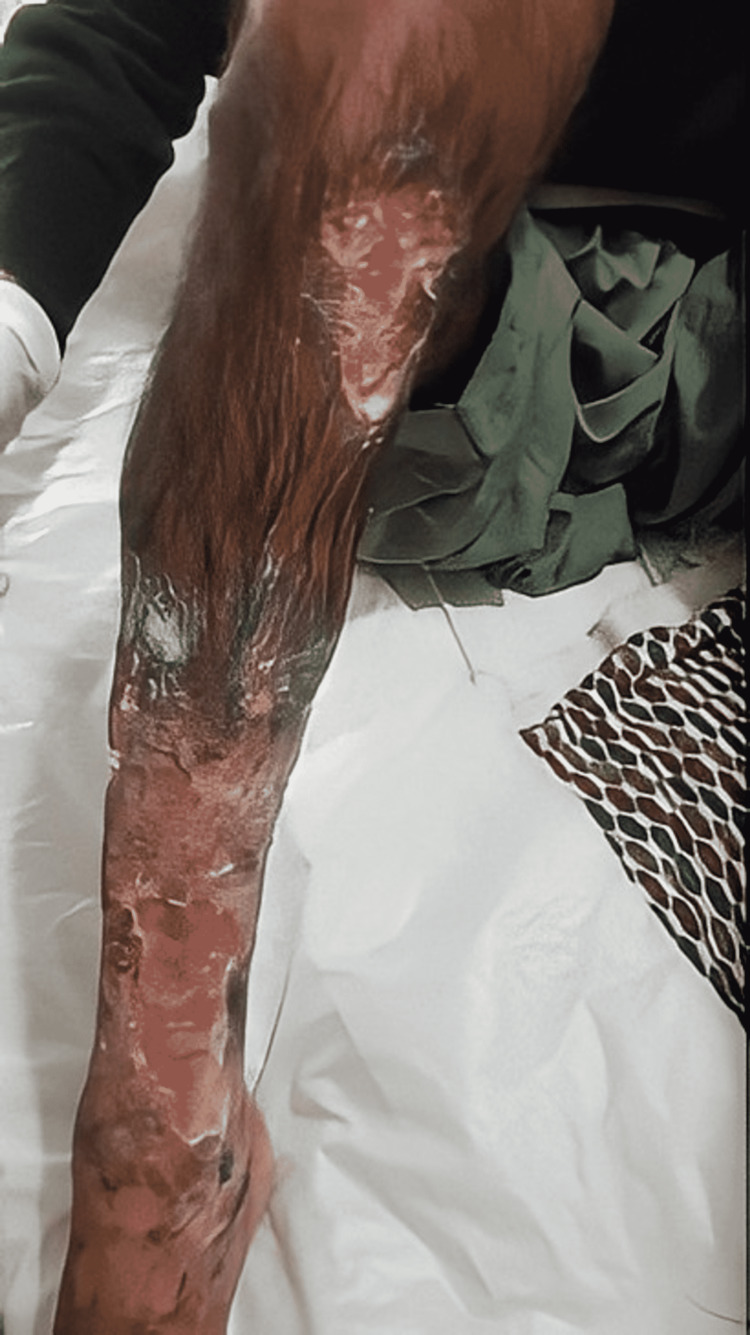
Right leg exhibits two large ulcers with undermined edges involving the extensor surface of the leg. Ulcers were formed following coalescence of multiple small ulcers. The picture was taken prior to the initiation of treatment.

**Figure 2:**
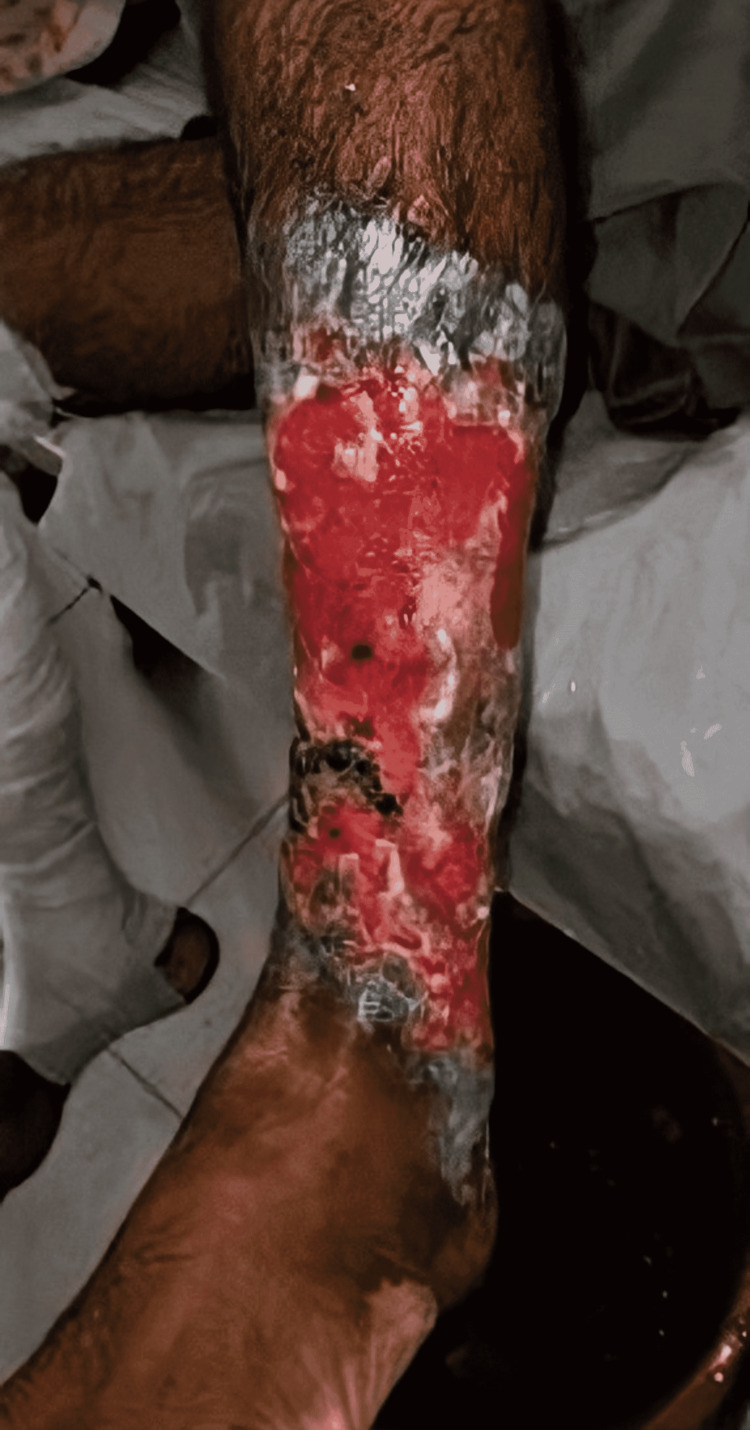
Left leg demonstrating a large, violaceous ulcer with undermined edges formed following coalescence of small ulcers. Bed of the ulcer stained with blood mixed with mucopurulent discharge is apparent. The picture was taken prior to the commencement of PG-targeted therapy. PG: pyoderma gangrenosum

A 35-year-old Asian male presented with bilateral, progressive, non-healing ulcers involving the shins for the past three months. The initial lesions on the extensor surface of the legs were erythematous papules that progressed to pustules and ultimately eroded and formed small, deep ulcers within the course of 10-15 days. Over time, the multiple small ulcers coalesced to form large, deep, non-healing ulcers (largest ulcer measuring 10 x 10 cm) associated with severe pain and itching, starting from the knees, and ultimately involving the extensor surfaces of the legs, and dorsum of both feet for the last three months of presentation (Figures 1, 2).

The patient had a prior history of hypertension and had been non-compliant with anti-hypertensive medications. He had not been diagnosed with diabetes, ischemic heart disease (IHD), asthma, or any other systemic diseases. He had not undergone any surgical procedures, nor did he have any prior hospital admissions. He had not sought any allopathic treatment for his ulcers and had instead resorted to herbal remedies, further aggravating his condition. There had been a history of application of occlusive dressings soaked in herbs, desi-ghee (oil), and rice on the small ulcers. This topical remedy was continued for two weeks, following which his condition worsened, reportedly. On general physical examination, pallor was noted, however, jaundice, edema, and cyanosis were absent. Evidence of lymphadenopathy, thyromegaly, and clubbing were also absent. The patient did not complain of any bowel or bladder-related problems in the weeks leading up to the formation of the ulcers. Detailed systemic examination was undertaken, identifying raised blood pressure (150/100 mmHg), however, no other abnormalities were observed. 

Upon admission to the hospital, he was treated empirically with antibiotics and analgesics, however, significant improvement was not observed. During workup, various investigations were carried out in an effort to rule out other causes of mucocutaneous ulceration. The medical workup for underlying infections only yielded a raised leucocyte count (24,500 cells/mm) and decreased hemoglobin levels (9.3 mg/dl). Other investigations were indicative of normal liver, renal and cardiovascular systems. Wound cultures were collected and demonstrated no evidence of microbial growth. Tuberculosis was effectively ruled out as well. Concurrently, there was an absence of arterial or venous insufficiency on the Doppler scan and detailed immunological studies were inconclusive at best as antinuclear antibody (ANA), rheumatoid factor (RF), anti-cyclic citrullinated peptide (anti-CCP), perinuclear anti-neutrophil cytoplasmic antibodies (p-ANCA), cytoplasmic anti-neutrophil cytoplasmic antibodies (c-ANCA) were negative. With ongoing symptomatic treatment of the ulcers, histopathology investigations on lesion biopsy showed central necrosis and ulceration of epidermis and dermis with neutrophilic infiltrates.

Besides the supportive treatment, debridement of the ulcers was carried out. However, rather than conferring relief, this further aggravated the ulcers. While being admitted in the medicine ward, the analgesics and antibiotics were administered intravenously and a new ulcer was noticed at the site of the cannulation on the fourth day of admission. This observation led us to believe that the ulcer was exhibiting the phenomenon of pathergy and our attention was diverted towards the spectrum of neutrophilic dermatoses. Ultimately, following the exclusion of other possible differential diagnoses, the patient was diagnosed as a case of pyoderma gangrenosum. A co-existing systemic disease could not be appreciated, therefore, it was a case of idiopathic pyoderma gangrenosum.

For the purpose of supportive treatment, non-adhesive moist dressings were employed. Saline-soaked gauze pieces were applied following which a loose crepe bandage was kept in place and the patient was advised to avoid trauma to the affected areas. Topical tacrolimus (0.1%) was applied twice daily to the affected area. The patient was due to be started on a combination of steroids (oral prednisolone 1 mg/kg) and cyclosporin. However, owing to the contraindication to cyclosporin by virtue of the patient being hypertensive, azathioprine was used as an adjunct to the steroid treatment.

**Figure 3:**
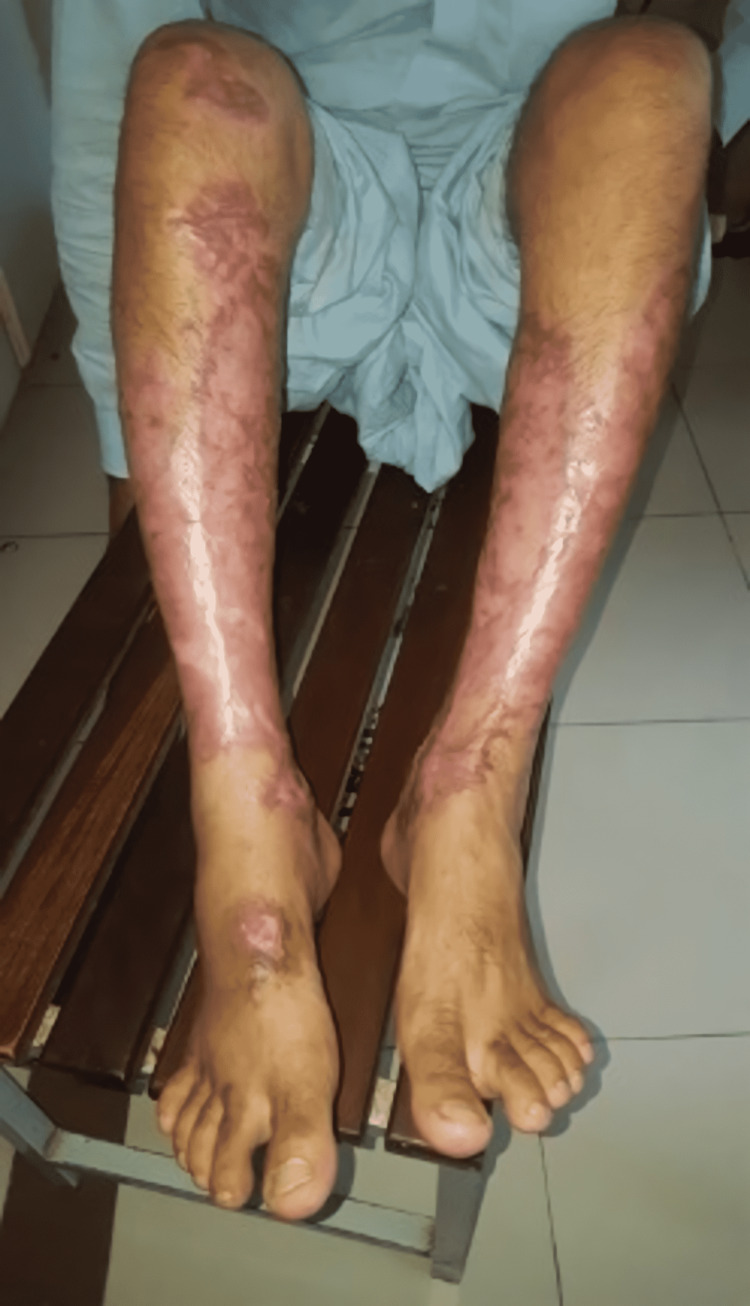
Healed ulcers on both legs showing characteristic cribriform scarring after three months of treatment with oral steroid (prednisolone) and azathioprine.

Thus, treatment was commenced with oral prednisolone (1 mg/kg, i.e., 50 mg in divided doses) and azathioprine (150 mg/day in divided doses). Prednisolone was tapered by 5 mg after two weeks of treatment initiation and was slowly tapered in the successive weeks. Alongside these, the patient was also prescribed an antihypertensive (losartan potassium - 50 mg once daily) to keep his blood pressure in check. Ulcers showed a rapidly promising response to this potent topical and systemic combination therapy, hence, this was continued with regular monitoring of his blood profile for the subsequent four months. The success of the treatment protocol employed was demonstrated with the healed cribriform pattern of scarring, documented three months from its commencement (Figure 3). Thus, remission was successfully attained in this patient. To our knowledge, this is the first time azathioprine has been employed as the primary adjunct in order to induce remission in a case of idiopathic pyoderma gangrenosum specifically among the Asian population.

## Discussion

Pyoderma gangrenosum is a rare skin disorder manifesting as cutaneous ulcers eroding the underlying skin. The estimated worldwide incidence of PG is three to 10 cases per million population per year, a fact that has been corroborated by prior retrospective studies and new-age multi-center, prospective studies [1]. The incidence of PG and its associated diseases varies in different populations. However, there is a paucity of antecedent medical literature on the subject specifically pertaining to the Asian population [2]. One case series of 18 patients documenting the rarity of PG in the Indian population has been published thus far [3]. On the contrary, in neighboring countries like Pakistan, there is a dearth of case reports and medical literature exploring the disease [4,5].

Fifty percent of the pyoderma gangrenosum cases are associated with an underlying disease, i.e., IBD, hematological malignancies, or they occur as part of inherited inflammatory syndromes. Thus, clinicians should investigate thoroughly for such conditions once they are suspecting PG [6]. Ulcers are preferentially located on lower limbs, however, upper limb involvement has been documented as well. Notably, pyoderma gangrenosum has a wide variety of clinical variants i.e it may manifest typically as the ulcerative type (commonest variant) or may occur as relatively less common atypical forms namely bullous, pustular, vegetative, peristomal, and extracutaneous [7].

Although the pathogenesis of pyoderma gangrenosum is not well understood, pre-existing literature on the topic is indicative of it being associated with the up-regulation of several cytokines. These cytokines include Interleukin 8 (IL-8), tumor necrosis factor (TNF), IL-1β, IL-17, and interferon gamma, among many others. TNF and IL-1β are of particular interest because some biological medications that target these cytokines have been effective in treating PG [8]. Thus, the underlying pathophysiology of the disease remains elusive for clinicians worldwide. Additionally, it does not have a specific appearance under the microscope, however, an abundance of white blood cells (neutrophils) has been observed in biopsy samples obtained from the ulcer edge. From a histopathological standpoint, classic pyoderma gangrenosum is characterized by neutrophilic infiltration of the dermis and destruction of the dermal tissue. However, variable findings depending on the clinical variant of pyoderma gangrenosum have been observed. Thus, biopsy confers a benefit in primarily excluding similar diseases exhibiting mucocutaneous ulceration [9]. 

The clinical course of pyoderma gangrenosum is usually characterized by an initial lesion in the form of either a papule, pustule, vesicle, or nodule that breaks down to form an erosion or ulcer. Fever may or may not be present. Pathergy test, i.e., the development of new skin lesions be it a papule or pustules, or the aggravation of existing ulcers following trivial trauma developing within 48 hours is positive in 31% of patients with pyoderma gangrenosum [10]. Ulcers ultimately heal with the classical* *pattern of “cribriform scarring*.*”

Diagnostic criteria for PG entail one major and eight minor criteria. Delphi exercise produced one major criterion, i.e., biopsy of ulcer edge demonstrating sterile neutrophilic infiltrate, and eight minor criteria, i.e., (1) exclusion of infection; (2) phenomenon of pathergy; (3) personal history of diseases characteristically associated with pyoderma gangrenosum, i.e., IBD, polyarthritis, myelodysplasia or leukemia (overall, 50% have a concomitant systemic illness); (4) history of papule, pustule, or vesicle ulcerating within four days of appearing; (5) peripheral erythema, undermined border, and eliciting tenderness at ulceration site; (6) multiple ulcerations, at least one on the anterior lower leg; (7) cribriform or "wrinkled paper" scar(s) at the site of healed ulcer; and (8) decreased ulcer size within one month of commencement of immunosuppressive medication(s) [11,12].

Thus, there are no specific existing tests to confirm a diagnosis of pyoderma gangrenosum. Diagnosis is based on a combination of clinical assessment, exclusion of other causes of skin ulceration, and certain investigations. Routine blood work to evaluate for an underlying systemic disease in a person suspected of being afflicted with pyoderma gangrenosum includes a complete blood count (CBC), a comprehensive chemistry profile, including a liver function test, renal function test, serum electrolytes, and a urinalysis. In addition, a hepatitis profile should be performed. Skin biopsy confers the benefit of effectively excluding other causes of skin ulceration and may ultimately divert the clinicians' attention toward the direction of neutrophilic dermatosis. Thus, careful exclusion of other disorders causing cutaneous ulceration after undertaking meticulous clinical examination and investigations is perhaps the first step in the management of the lesions that appear consistent with pyoderma gangrenosum.

Owing to the relative scarcity of data on interventions, definitive guidelines for the diagnosis and management are lacking in the case of pyoderma gangrenosum. The choice of treatment modality is based on the severity and extent of the disease. The management approach to PG is primarily guided by small, uncontrolled studies and clinical experience. In general, patients are managed with a combination of topical and/or systemic therapies which suppress the inflammatory process and wound care measures that facilitate an optimal wound healing environment. Although initial signs of improvement may be evident within days of the start of treatment, weeks to months are often required to achieve complete ulcer healing and remission.

Supportive therapy with appropriate dressings, compression (following exclusion of arterial ulcers), and adequate analgesia are all essential to optimize the process of healing. Among the topical treatments, topical tacrolimus (0.1%) is the most commonly utilized treatment modality. Potent topical corticosteroids along with tacrolimus ointment applied to the ulcer surface are particularly of benefit. Intralesional injections of corticosteroids into the erythematous active border may also be considered as well [11]. Corticosteroids are the mainstay of systemic treatment and are utilized to gain rapid control over the disease. Cyclosporin can be used either in combination with corticosteroids or alone as a steroid-sparing agent, in cases where prolonged treatment is required [13]. Therefore, both are used in conjunction as first-line agents in order to gain control over systemic disease and may confer an equal benefit in minimizing the ulcerative process and inducing remission [14].

Second-line agents include immunosuppressants such as methotrexate, cyclophosphamide, chlorambucil, tacrolimus, mycophenolate, and azathioprine. These agents have been known to be advantageous in the management of PG, however, they are mostly utilized as adjuncts. Combination immunosuppressant therapy may be a treatment option for patients with severe, rapidly progressive pyoderma gangrenosum [15]. Biological agents such as infliximab, adalimumab, etanercept among others have been shown to exert a beneficial effect in certain patients with poor response or recalcitrant pyoderma gangrenosum [16].

## Conclusions

Pyoderma gangrenosum is a rare, non-contagious, inflammatory neutrophilic dermatosis that may have a protracted clinical course owing to delay in its diagnosis and management. Early diagnosis of the disease remains crucial in minimizing its inflammatory course. It is imperative to exclude other diseases with similar patterns of mucocutaneous ulceration, first and foremost while taking the help of histopathology results in the process. Initiation of treatment with topical and systemic agents must be employed at the earliest when a diagnosis of pyoderma gangrenosum is suspected. Although corticosteroids and cyclosporin remain the mainstay for the treatment of the disease. The efficacy of second-line immunosuppressants such as azathioprine must be explored as there are limited pre-existing case reports and controlled studies on the subject, primarily in developing Asian countries where biological agents are not commonly utilized or generally inaccessible.
